# Supplementary material for: Agent-based modelling of reactive vaccination of workplaces and schools against COVID-19
Source: Nat Commun. 2022 Mar 17;13:1414. doi: 10.1038/s41467-022-29015-y (PMC8931017; doi:10.1038/s41467-022-29015-y)
Supplement: Supplementary file 1 — Supplementary Information [file 41467_2022_29015_MOESM1_ESM.pdf]

Supplementary Information

Agent-based modelling of reactive vaccination of workplaces and schools against COVID-19

Benjamin Faucher[1], Rania Assab[1], Jonathan Roux[2], Daniel Levy-Bruhl[3], Cécile Tran Kiem[4,5], Simon Cauchemez[4], Laura Zanetti[6], Vittoria Colizza[1,7], Pierre-Yves Boëlle[1], Chiara Poletto[1]

[1]Sorbonne Université, INSERM, Institut Pierre Louis d'Epidémiologie et de Santé Publique, F75012, France

[2]Univ Rennes, EHESP, CNRS, ARENES – UMR 6051, F-35000 Rennes, France

[3]Santé Publique France, Saint Maurice, France

[4]Mathematical Modelling of Infectious Diseases Unit, Institut Pasteur, Université de Paris, UMR2000, CNRS, Paris, France

[5]Collège Doctoral, Sorbonne Université, Paris, France

[6]Haute Autorité de Santé, Saint-Denis, France

[7]Tokyo Tech World Research Hub Initiative (WRHI), Tokyo Institute of Technology, Tokyo, Japan

Corresponding author: Chiara Poletto (chiara.poletto@inserm.fr)

Supplementary Methods 2

1. COVID-19 transmission model 2
2. Test-trace-isolate 4
3. Vaccination strategies 6
4. Details on the epidemic simulations 8

Supplementary Note 1: Comparison between reactive and non-reactive vaccination strategies 11

1. Distribution of the attack rates 11
2. Vaccinated settings 12
3. Sensitivity analysis 13
4. Additional epidemic outcomes 17

Supplementary Note 2: Combined reactive and mass vaccination for managing sustained COVID-19 spread 21

1. Additional results 21

Supplementary Note 3: Combined reactive and mass vaccination for managing a COVID-19 flare-up 21

1. Additional results 21
2. Sensitivity analysis 22

Supplementary References 24

Supplementary Methods

1. COVID-19 transmission model

We provide here in the following the parameter values for transmission and infection natural history (Supplementary Table 1), and the effect of vaccination (Supplementary Table 2). For a detailed explanation of the transmission model without vaccination we refer to[1]. Incubation period IP and length of the pre-symptomatic phase μ_p are specific for the Delta variant. In particular, μ_p is parametrised based on the proportion of pre-symptomatic transmission estimated in [2].

Supplementary Table 1. Transmission parameters and their baseline values.

Parameter	Description	Baseline value (other explored values)	Source
IP	Incubation period	5.8 days (5.1 days, 6.3 days)	[2] ([3,4])
$(\mu_p)^{-1}$	Average duration of the pre-symptomatic stage	2.1 days	Computed to recover 74% of pre-symptomatic transmission as estimated in [2]
$(\epsilon)^{-1}$	Rate of becoming infectious for exposed individuals	3.7 days	$IP - \mu_p^{-1}$
μ	Recovery rate	$(7 \text{ days})^{-1}$	[5]
β_I	transmissibility rescaling according to the infectious stage	0.51 for $I_{p,sc}$, I_{sc}<br>1 for $I_{p,c}$, I_c	[6]
ω_s	Transmission risk by setting	1 for household<br>0.3 for community<br>0.5 otherwise	[1]

Supplementary Table 2. Vaccine effectiveness parameters and their baseline values.

Parameter	Description	Baseline value (other explored values)	Source
$r_{S,1}$	reduction in susceptibility in the partial-protection stage	0.52 (0.35, 0.7)	from $VE_{S,1} = 48\%$, in the middle of the range of estimates after one dose in [7] (from 30% and 65%, worst and best estimates from [7], respectively)
τ_0	Average duration of the no-protection stage after first-dose inoculation	2 weeks (1 week)	
$r_{S,2}$	reduction in susceptibility in the maximum-protection stage	0.3 (0.2, 0.47)	from $VE_{S,2} = 70\%$, in the middle of the range of estimates after two doses in [7] (from 53% and 80%, worst and best estimates from [7], respectively)
$r_{c,2}$	reduction in the probability of developing clinical symptoms in the maximum-protection stage	0.9 (0.4, 0.95)	from $VE_{SP,2} = 73\%$, in the middle of the range of estimates after two doses in [7] (from 60% and 95%, worst and best estimates from [7], respectively)
τ_1	Average duration of the intermediate-protection stage	3 weeks (4 week, 8 week)	[8]
p_V	Probability of transition between $S^{V,1}$ and E^V	0.97 (0.65, 0.82)	Computed from the parameters above and assuming $VE_{SP,1} = 53\%$, in the middle of the range of estimates after one dose in [7] (from 35%, 75%, worst and best estimates from [7], respectively) (*)
r_I	Reduction in infection duration	25% (0)	[9]

(*) Mid range estimate in [7], $VE_{SP} = 55\%$, leads to a value of $p_V > 1$, thus $VE_{SP} = 53\%$ is taken.

2. Test-trace-isolate

We model case detection and isolation, combined with tracing and isolation of contacts according to the following rules:

- As an individual shows clinical symptoms, s/he is detected with probability $p_{d,c}$. If detected, case confirmation and isolation occur with rate r_d upon symptoms onset.
- Subclinical individuals are also detected with probability $p_{d,sc}$, and rate r_d .
- The index case's household contacts are isolated, with probability $p_{ct,HH}$, the same time the index case is detected and isolated. We assume that these contacts are tested at the time of isolation and among those all subclinical, clinical, pre-subclinical, and pre-clinical cases are detected (testing sensitivity 100%).
- Once the index case is detected, contacts of the index case occurring outside the household are traced and isolated with an average delay r_{ct}^{-1}. We define an acquaintance as a contact occurring frequently, i.e. with a frequency of activation higher than f_a. We assume that an acquaintance is detected and isolated with a probability $p_{ct,A}$, while other contacts (i.e. sporadic contacts) are detected and isolated with probability $p_{ct,sp}$, with $p_{ct,A} > p_{ct,sp}$. We assume that traced contacts are tested at the time of isolation and among those all subclinical, clinical, pre-subclinical, and pre-clinical cases are detected (testing sensitivity 100%).
- Only contacts (among contacts occurring both in household and outside) occurring within a window of D days before index case detection are considered for contact tracing.
- The index-case and the contacts are isolated for a duration d_I (for all infected compartments) and d_{NI} (for susceptible and recovered compartments). Contacts with no clinical symptoms have a daily probability p_{drop} to drop out from isolation.
- For both the case and the contacts, isolation is implemented by assuming no contacts outside the household and transmission risk per contact within a household reduced by a factor ι.

Parameter values are reported in Supplementary Table 3 and Supplementary Table 4 for baseline and enhanced TTI, respectively.

Supplementary Table 3. Model for test, trace, isolation. Parameters and their values for the baseline case.

Parameter	Description	Value	Source
$p_{d,c}$	Probability that a clinical case is detected	0.5	
$p_{d,sc}$	Probability that a subclinical case is detected	0.1	
r_d	For detected cases, rate of detection, confirmation and beginning of isolation	0.28 = (3.6 days)$^{-1}$	Average time from onset to testing is 2.6 days [10]. We assume one day to have the test results.
D	Length of the period preceding index-case confirmation, used to define a contact	6 days	$\simeq$2 days + ${r_d}^{-1}$ (a person is considered to be contact if s/he entered in contact with the index case during a window of 2 days preceding symptoms onset)
$p_{ct,HH}$	Probability that household contacts of an index case are identified and decide to isolate	0.7	
$p_{ct,A}$	Probability that acquittances of an index case are identified and decide to isolate	0.08	Calibrated to get $\simeq$2.8 contacts per index case on average (assumed $p_{ct,Oth} = p_{ct,A}/10$) [10]
$p_{ct,Oth}$	Probability that sporadic contacts of an index case are identified and decide to isolate	0.008	Calibrated to get $\simeq$2.8 contacts per index case on average (assumed $p_{ct,Oth} = p_{ct,A}/10$) [10]
r_{ct}	Rate of detection and isolation of contacts outside household	0.9 = (1.1 days)$^{-1}$	
f_a	Threshold frequency to define a contact as an acquaintance	1/7 days	
p_{do}	Probability to drop out from isolation for individuals that are not clinical	0.13	[11]
ι	Reduction in household transmission during isolation	0.5	

d_{NI}, d_I	Duration of isolation for an individual that is not infectious, duration of isolation for an individual that is infectious	10 days, 10 days	12

Supplementary Table 4. Model for test, trace, isolation. Parameters and their values for the case of enhanced TTI. Only values different from the baseline case are reported.

Parameter	Description	Baseline value
$p_{d,c}$	Probability that a clinical case is detected	0.7
$p_{d,sc}$	Probability that a subclinical case is detected	0.3
r_d	For detected cases, rate of detection, confirmation and beginning of isolation	0.9 = (1.1 days)$^{-1}$
$p_{ct,HH}$	Probability that household contacts of an index case are identified and decide to isolate	1
$p_{ct,A}$	Probability that acquittances of an index case are identified and decide to isolate	0.24
$p_{ct,Oth}$	Probability that sporadic contacts of an index case are identified and decide to isolate	0.024
p_{do}	Probability to drop out from isolation for individuals that are not clinical	0

3. Vaccination strategies

We provide here in the following the parameters values for the vaccination strategies detailed in the Methods section of the main paper.

Supplementary Table 5. Vaccine administering. Parameters and their baseline values.

Parameter	Description	Baseline values (other explored values)
$a_{th,V}$	Minimum age for vaccination	12 years
P_V^a	Probability an individual is willing to vaccinate	90% for 65+<br><br>80% for <65 (60%, 100%)
n_{cl}	For reactive vaccination, cluster size for triggering reactive vaccination in a workplace or school	1 (2, 3, 4, 5)
T_{cl}	For reactive vaccination, time window for cluster definition	7 days
$(r_V)^{-1}$	For reactive vaccination, delay of implementation of the vaccination campaign once the cluster is identified (for workplaces/schools) and a case is identified (for households)	2 days (1 day, 4 days)
V_{TOT}	Maximum number of people vaccinated during one simulation	Unlimited
V_{day}	Maximum number of people vaccinated each day	Explored in the range [50, 500] per 100,000 inhabitants per day for mass, workplaces/universities, school locations<br><br>Unlimited for reactive (explored in [50, 250] per 100,000 inhabitants per day)
$size_{th}$	For workplaces/universities, minimum size of a workplace that implement vaccination	20
n_{RV}	For reactive vaccination, cluster size for starting the reactive vaccination campaign in the flare up scenario	1, 5, 10
	Initial vaccination coverage	90% for 65+<br><br>40% for <65 (15%, 25%, 35%, 45%, 55%, 65%, 75%)

4. Details on the epidemic simulations

A schematic representation of the main program and of the simulation code and of the algorithm used for a single stochastic realisation are shown in Supplementary Figure 1 and 2, respectively. Simulations are discrete-time and stochastic. At each time step, corresponding to one day, three processes occur (Supplementary Figure 2):

Vaccination Step: vaccines are administered according to the strategy or the strategies' combination.

Testing Step: cases are detected and isolated; contacts (within and outside household) are identified and isolated; isolated individuals get out from isolation.

Transmission Step: infectious status of nodes is updated. This includes transmission, recovery and transition through the different stages of the infection (e.g. from exposed to pre-symptomatic, from pre-symptomatic to symptomatic).

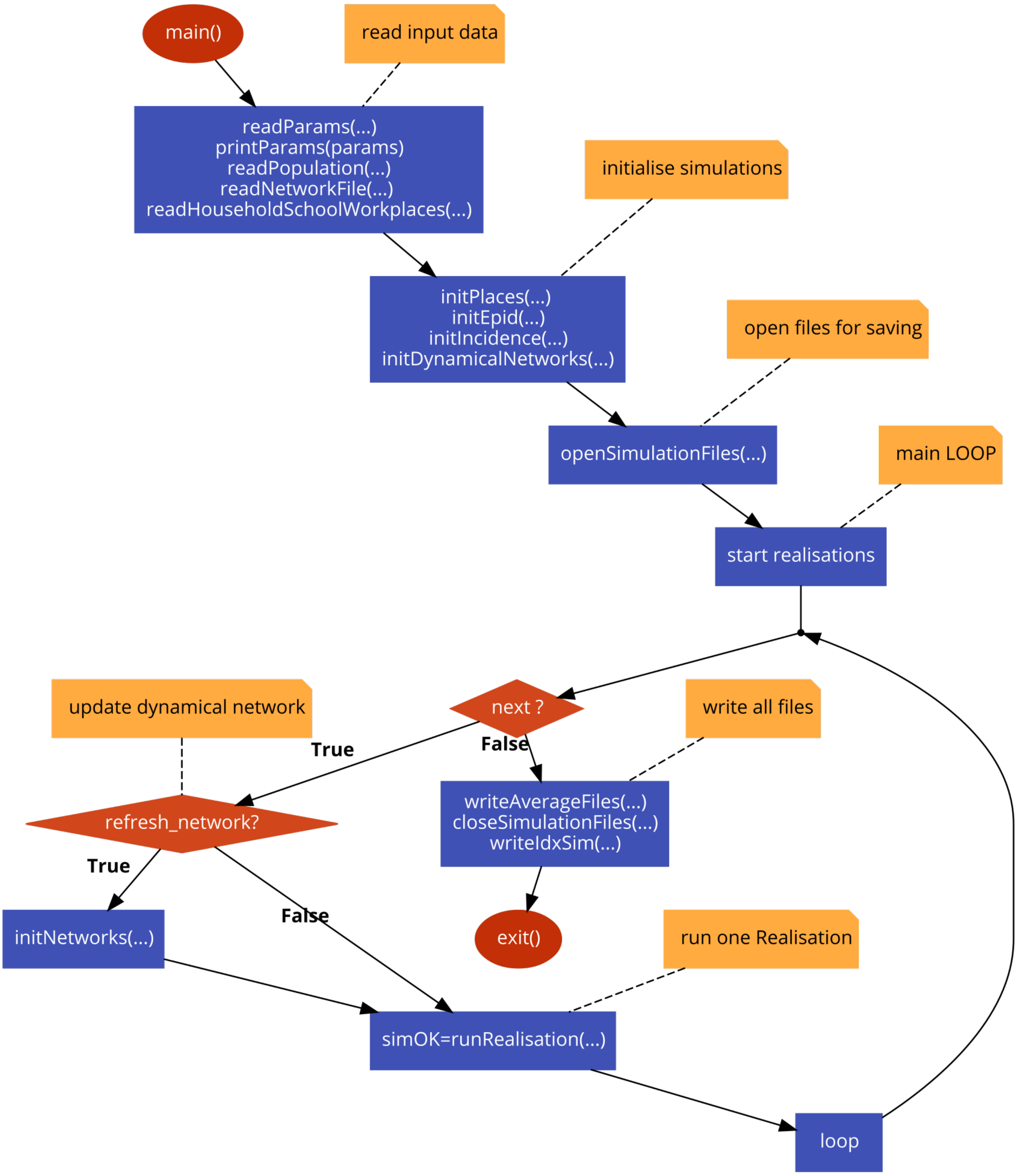

Supplementary Figure 1: Algorithm of the main program. Algorithm of the main program drawn with code2flow.com.

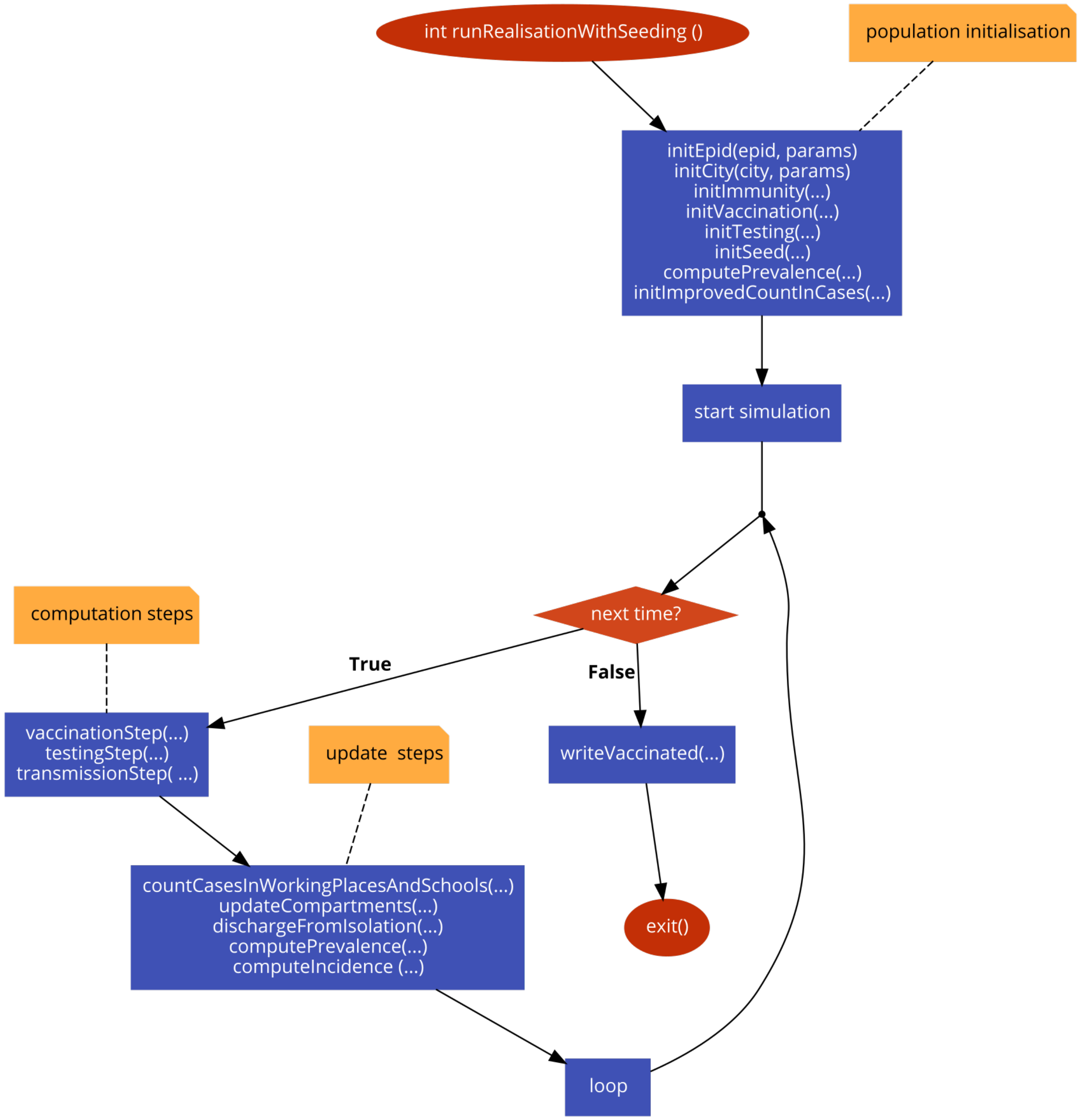

Supplementary Figure 2: Algorithm for one stochastic realisation. Algorithm for one stochastic realisation drawn with code2flow.com.

A single-run simulation is executed with no modelled intervention, until the desired immunity level is reached. This guarantees that immune individuals are realistically clustered on the network. We added some noise, by reshuffling the immune/susceptible status of 30% of the nodes to account for travelling, infection reintroduction from other locations and large gathering with consequent super-spreading not accounted for by the model. In Fig. 2 and 3 of the main

paper, all processes (transmission, TTI, vaccination) are simulated from the beginning of the simulation. In the flare-up scenario (Fig. 4), TTI and mass vaccination are modelled from the beginning. TTI is enhanced from the detection of the first case. Reactive vaccination starts after the detection of the first n_{RV} cases, with $n_{RV} = 1,5,10$ explored.

We vary COVID-19 transmission potential by tuning the daily transmission rate per contact β. The reproductive number R is computed numerically as the average number of infections each infected individual generates throughout its infectious period at the beginning of the simulation. Therefore, it integrates the effect of the interventions and the level of disease and vaccine induced immunity in the population at the start. We tune β to have the desired R value for the reference scenario, i.e. with only vaccination at the start. We then compare different vaccination strategies at the same value of transmissibility β.

To calibrate the duration of the pre-symptomatic stage from [2] (value reported in Supplementary Table 1) we generated an output file containing the list of all transmission events with the infection status of the infector. The proportion of pre-symptomatic transmission was computed as the fraction of transmission events with infector in the compartment $I_{p,sc}$ or $I_{p,c}$ over all infection events.

Supplementary Note 1: Comparison between reactive and non-reactive vaccination strategies

1. Distribution of the attack rates

We compare here the distributions of the attack rates after two months across different strategies. In the Supplementary Figure 3, we consider the scenarios of Fig. 2e of the main paper and we show that the distribution and the standard deviation are similar among vaccination strategies.

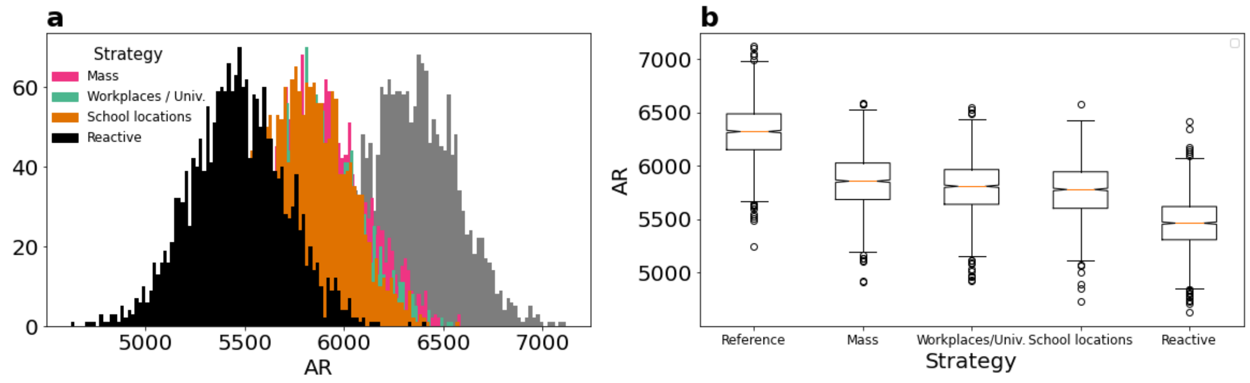

Supplementary Figure 3: Distribution of the attack rate with different vaccination strategies. a Distribution of attack rates after two months for the scenarios analysed in Fig. 2e of the main paper. **b** Boxplots comparing the distributions of attack rates after 2 months for different vaccination strategies. Medians are represented by medium red lines and interquartiles (Q1-Q3) by boxes. The whiskers delimit the range between Q1 - 1.5*IQR and Q3 + 1.5*IQR. Outside values are considered as outcomes and are represented by points.

2. Vaccinated settings

We provide here a detailed analysis of the time evolution of the number of settings where vaccines are deployed in the context of reactive vaccination.

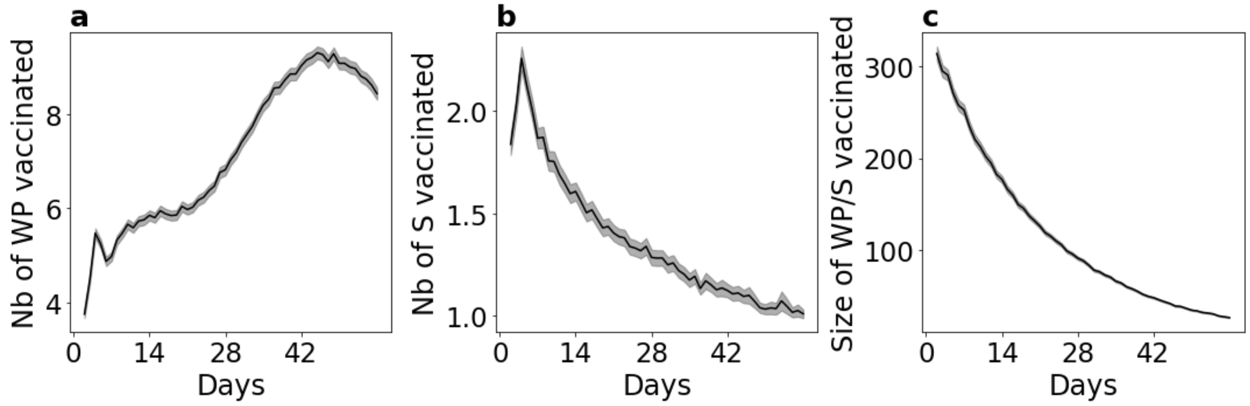

Supplementary Figure 4: Details analysis of settings where vaccines were deployed in the context of reactive vaccination. a, b Daily number of workplaces (**a**) and schools (**b**) where vaccines are deployed in the context of reactive vaccination. **c** Size of workplaces/Schools where vaccines are deployed as a function of time. Parameters are the same as in Fig. 2c of the main paper. In all panels continuous lines are means over 2000 independent stochastic realisations and the shaded areas are the standard error of the mean (+/- 2SEM).

3. Sensitivity analysis

We compare here reactive vaccination with non-reactive vaccination strategies under a variety of epidemic scenarios.

In Supplementary Figure 5 we compare all strategies at equal number of doses over the two-months period, exploring the impact of the following parameters: reproductive ratio, immunity level of the population, repartition of contacts across settings due to social distancing, incubation period, effect of the vaccine in the infection duration, vaccine uptake, and time between doses. Except when otherwise indicated, parameters are the ones of the baseline scenario with intermediate vaccination coverage (~45% of the whole population vaccinated, Fig. 2 d, e of the main paper). Increasing the transmissibility or initial immunity reduces the impact of reactive vaccination (panel a, b). In panel c we explore the impact of teleworking and reduction in community contacts by comparing the baseline scenario with scenarios with no or stronger restrictions. Reactive vaccination becomes more effective when no restriction is in place - although the effect is not strong. This is likely due to the enhanced role of workplaces as a setting of transmission when no teleworking is in place, thus bringing to an increased benefit of reactive vaccination targeting this setting. In panel d, we analyse the impact of the choice of the incubation period exploring values ranging from 5.1 [3] up to 6.3 [4]. We find that results are highly robust to the choice of the parameters within this range. In panel e we compare different hypotheses regarding the effect of the vaccine on the infection duration, i.e. the baseline case with the case in which the vaccine induces no reduction in the infectious duration. Also in this case, results are robust. Eventually in panel f we compare different levels of vaccine uptake - assuming uptake to be the same in the reactive and non-reactive strategies as in Fig. 2 of the main paper. The impact of vaccination increases with the uptake, the effect being stronger for the reactive strategy. Eventually, we compare in panel g different timing for vaccine-induced immunity to become effective. Specifically, we consider a case in which partial protection against infection mounts one week after the first dose. Assuming an incubation period of ~6 days, this would be consistent with no reduction in cases observed ~2 weeks after first-dose inoculation, as reported by some real vaccine effectiveness studies [13,14]. We then consider a longer interval between doses (i.e. 4 and 8 weeks). Assuming that protection against infection starts one week after first-dose inoculation leads to a higher impact of vaccination for all four vaccination strategies.

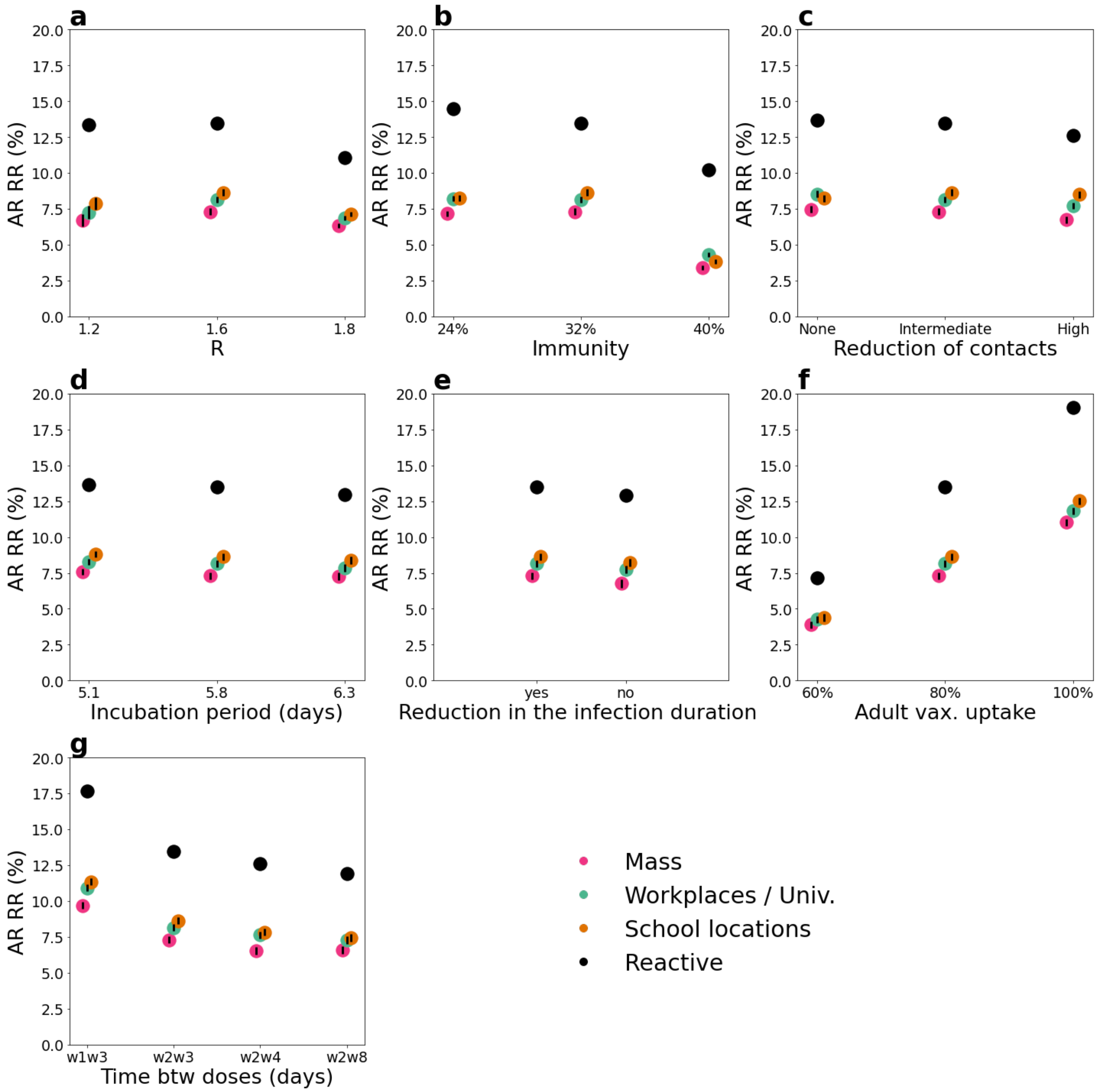

Supplementary Figure 5: Comparison between vaccination strategies - sensitivity analysis. Relative reduction (RR) in the attack rate (AR) after two months for all strategies at equal number of doses. RR is computed with respect to the reference scenario with initial vaccination only. Different parameter values and modelling assumptions are compared. Vaccination rate for mass, workplaces/universities and school location vaccination is set to the average value recovered for reactive vaccination. Exceptions made for the parameter explored in each panel - indicated in the x-axis -, all parameters are as in Fig. 2e. Parameters explored are: **a** reproductive ratio; **b** natural immunity of the population at the start; **c** repartition of contacts across settings due to social distancing (*Intermediate* is the baseline scenario, while *high* is given by a 30% reduction in community contacts and a 20% of individuals doing teleworking); **d** incubation period, **e** vaccine-induced reduction in infection duration (yes, no), **f** vaccine uptake, and **g** time between doses - the x-axis labels wNwM indicates the average number of weeks, N and M, of no protection following

first dose inoculation and of intermediate vaccine effectiveness, respectively. In all panels data are represented as means over 2000 independent stochastic realisations and error bars are derived from the standard error of the mean. These are smaller than the size of the dots in almost all cases.

The impact of reactive vaccination and its demand in terms of vaccine doses varies depending on the incidence level. In Supplementary Figure 6a-c we compare all strategies under a scenario of flare-up of cases. All parameters are as in the baseline scenarios, intermediate vaccination coverage (~45% of the whole population vaccinated, Fig. 2 d, e of the main paper), except for initial incidence. Here we assumed three cases were infected at the beginning. Panel a shows the relative reduction in the attack rate after two months as a function of the number of first daily doses, while panel b compares the incidence profiles under different strategies at equal number of vaccine doses. The relative reduction produced by reactive vaccination is close to the one produced by mass, school location and workplaces/universities. Panel c shows the number of vaccines deployed each day for reactive vaccination and the number of workplaces/schools where vaccines are deployed. These are initially low and increase gradually with incidence.

We then explore the impact of the reactive vaccination in the flare-up case in varying the different parameters. Specifically, we compare all strategies at an equal number of doses, varying the level of social distancing (Supplementary Figure 6d) and the timing for the immunity to mount after the first-dose vaccination (Supplementary Figure 6e). For certain parameter values reactive vaccination produces a higher relative reduction in the attack rate compared with non-reactive strategies. This is the case for instance when the development of vaccine immunity is rapid, and when no social restrictions are in place. In other cases it produces comparable effect. This is the case for instance of long delays between doses. Finally, in Supplementary Figure 6f, we provide an overview of the impact of initial incidence. As initial incidence increases the advantage of the reactive vaccination compared with the non-reactive strategies increases.

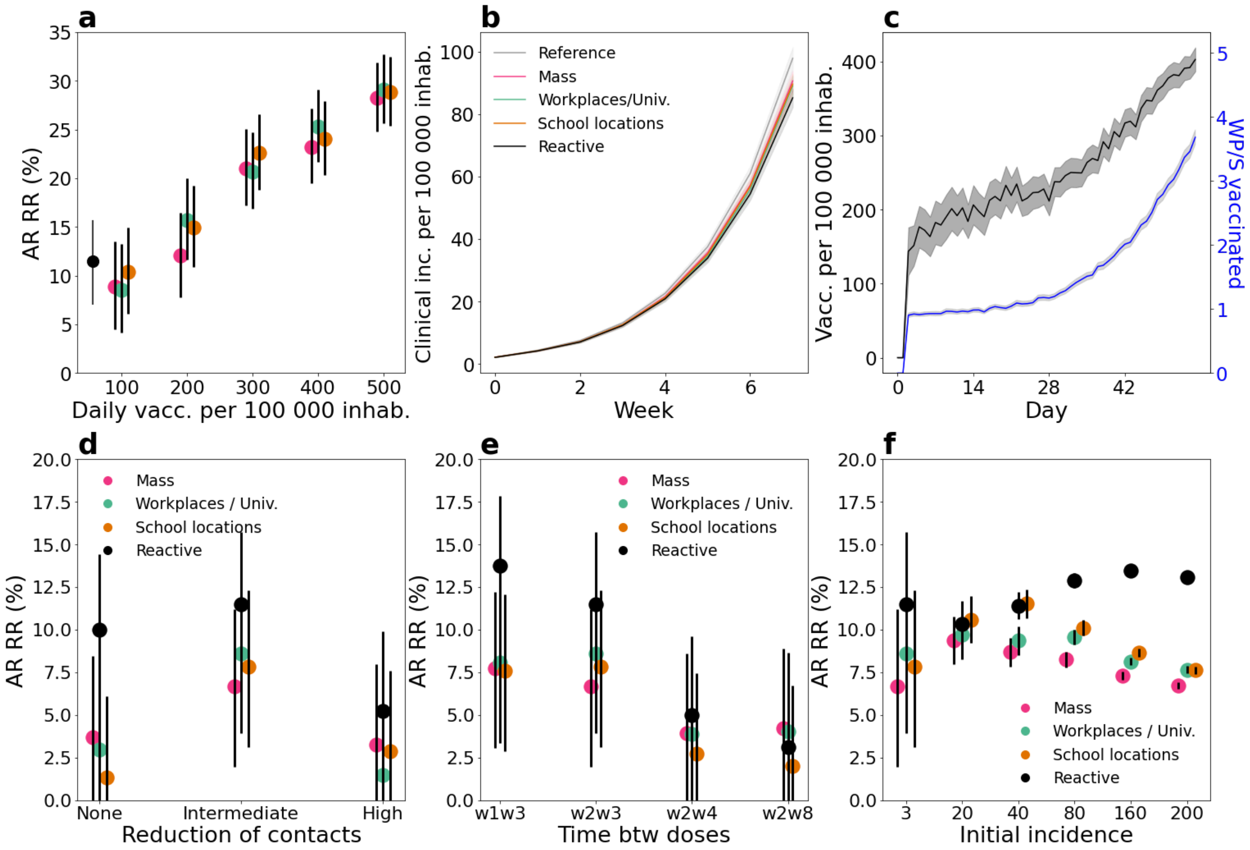

Supplementary Figure 6: Comparison between vaccination strategies - flare-up scenario. a Relative reduction (RR) in the attack rate (AR) over the first two months for all strategies as a function of the vaccination pace. RR is computed with respect to the reference scenario, with initial vaccination only. **b** Incidence of clinical cases with different vaccination strategies during the first 8 weeks. **c** Number of daily first-dose vaccinations, and workplaces/schools (WP/S in the plot) where vaccines are deployed. **d** AR RR after two months according to the repartition of contacts across settings due to social distancing - *Intermediate* is the baseline scenario, while *high* is given by a 30% reduction in community contacts and a 20% of individuals doing teleworking. **e** AR RR after two months according to the timing for the immunity to mount after first-dose vaccination - the x-axis labels wNwM indicates the average number of weeks, N and M, of no protection following first dose inoculation and of intermediate vaccine effectiveness, respectively. **f** AR RR after two months according to the initial incidence for all strategies at equal number of doses. In panels **b,d-f** all strategies are compared at equal number of doses. All panels, except for panel **f**, consider a flare up scenario, where the epidemic is seeded with 3 infectious individuals. All other parameters are as in Fig. 2d, e of the main paper. In panels **a**, **d-f** data are represented as means over 2000 independent stochastic realisations and error bars are derived from the standard error of the mean. In panels **b**, **c** continuous lines are means over 2000 independent stochastic realisations and the shaded areas are the standard error of the mean (+/- 2SEM).

4. Additional epidemic outcomes

Based on the estimated incidence of clinical cases per day provided by the transmission model, we infer outcomes related to hospital, namely hospital and intensive care unit (ICU) entries, beds in ICU ward, and deaths. We use age-dependent hospital admissions (ICU and non-ICU) risks estimated by [15,16] and ICU admission risks for hospitalised patients based on SI-VIC extract [17]. Hospital admissions risks were adjusted to apply only to clinical cases [18] and to account for vaccine effectiveness for hospitalisation for zero, half (1 dose) and full (2 doses) vaccination. We assume the vaccine efficacies for hospitalisation were equal to 83% and 94% for half and full vaccinations, respectively[7]. We also assume that the hospital admission risk increases by 80% with the Delta variant compared to Alpha [19] and by 40% with the Alpha variant compared to the wild type [20]. Patients who were hospitalised entered the hospital on average 7 days (sd = 3.9 days – Gamma distribution) after the beginning of the infectious phase [21]. Those who were admitted in ICU enter this unit with a mean delay of 1.69 days (assuming an exponential distribution) [17]. To estimate the number of occupied beds, we use age-specific mean durations of stay and their corresponding standard deviations in ICU calculated on all the hospitalised cases in the first 9 months of the French epidemic (March-November 2020)[17]. We assume that the standard deviations of ICU lengths of stay were equal to the corresponding mean and do not consider post-ICU care in the estimation of occupied beds. We estimate the number of deaths using hospital and ICU death risks of hospitalised infected persons [17]. Deaths are delayed in time using the mean delays and standard deviations from hospital or ICU admission to death [17]. All lengths of stay are supposed to follow a Gamma distribution. Parameters and their values are summarised in Supplementary Tables 6 and 7.

We also estimate the number of life years and quality-adjusted life years (QALY) lost for each death using life-tables provided by ‘French National Institute of Statistics and Economic Studies’ (INSEE) for 2012-2016 [22] and utility measures of each age-group in France [23].

Supplementary Table 6. Risks of hospitalisation according to vaccination status, ICU admission and death in general ward and ICU

Age group	Hospitalisation risk for no-vaccination	Hospitalisation risk for 1-dose vaccination	Hospitalisation risk for 2-doses vaccination	ICU admission risk	Death risk in general ward	Death risk in ICU
0-9	0.0246	0.0115	0.0066	0.159	0.006	0.078
10-19	0.0136	0.0063	0.0037	0.159	0.006	0.078
20-29	0.0364	0.0170	0.0098	0.159	0.006	0.078
30-39	0.0443	0.0207	0.0119	0.159	0.006	0.078
40-49	0.0617	0.0288	0.0166	0.219	0.016	0.103
50-59	0.1697	0.0792	0.0457	0.270	0.030	0.175
60-69	0.2360	0.1101	0.0635	0.299	0.064	0.268
70-79	0.5113	0.2386	0.1377	0.235	0.140	0.366
79+	0.9496	0.4431	0.2557	0.053	0.308	0.468

Supplementary Table 7. Delays in days from hospitalisation admission in general ward to death or hospital discharge and delays from ICU admission to ICU discharge or death.

Age group	Mean los in general ward (sd)	Mean los in general ward for dying people (sd)	Mean los in ICU (sd)	Mean los in ICU for dying people (sd)
0-9	6.4 (6.8)	10.6 (12.3)	12.7 (12.7)	22.3 (22.3)

10-19	6.4 (6.8)	10.6 (12.3)	12.7 (12.7)	22.3 (22.3)
20-29	6.4 (6.8)	10.6 (12.3)	12.7 (12.7)	22.3 (22.3)
30-39	6.4 (6.8)	10.6 (12.3)	12.7 (12.7)	22.3 (22.3)
40-49	6.4 (6.8)	10.6 (12.3)	12.7 (12.7)	22.3 (22.3)
50-59	6.4 (6.8)	10.6 (12.3)	12.7 (12.7)	22.3 (22.3)
60-69	9.3 (9.1)	10.6 (12.3)	16.7 (16.7)	20.8 (20.8)
70-79	11.7 (11.4)	10.6 (12.3)	17.5 (17.5)	18.9 (18.9)
79+	15 (13.8)	10.6 (12.3)	13.6 (13.6)	10.6 (10.6)

Los: Length of stay. Sd = Standard deviation.

Supplementary Figure 7 shows the relative reductions in the number of hospitalisations, deaths, ICU entries, life-year lost, quality-adjusted life-year lost and ICU bed occupancy at the peak, comparing each vaccination scenario with the reference scenario - i.e. vaccination only at the start. We consider here the high incidence and intermediate vaccine coverage scenario, analogously to Fig. 2d, e of the main paper. The six indicators show a behaviour similar to incidence. Overall reduction values are smaller. This is expected, since a large proportion of elderly are already vaccinated at the start, and the compared vaccination strategies target a population that is less at risk of severe infection. Still, all indicators show the same qualitative behaviour, with reactive vaccination outperforming the non-reactive vaccination strategies at equal number of first-dose vaccination.

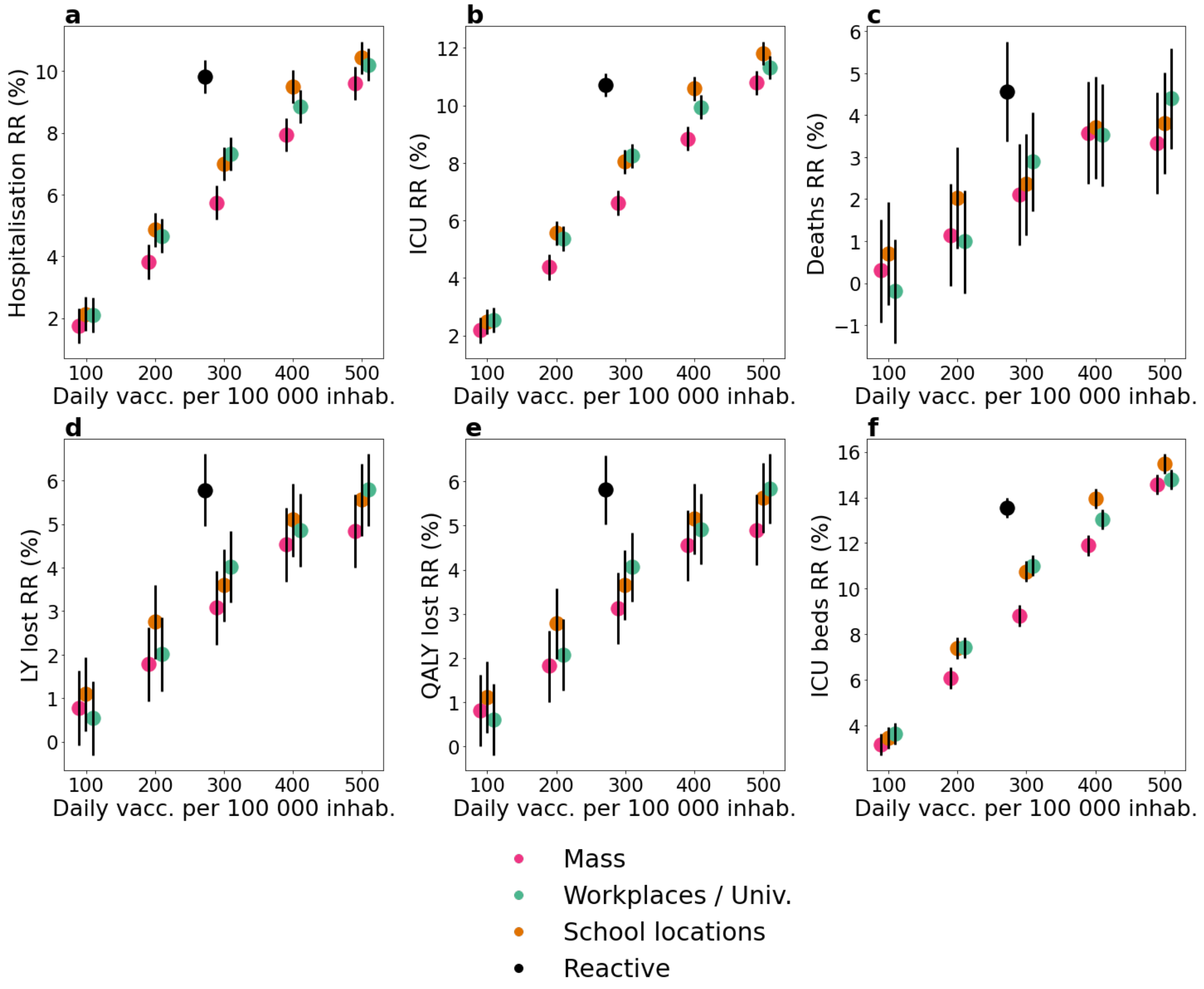

Supplementary Figure 7: Comparison between vaccination strategies - additional epidemic outcomes. Relative reduction (RR) in the cumulative incidence of: **a** hospitalisations, **b** intensive care unit (ICU) entries, **c** deaths, **d** life years (LY) lost and **e** quality-adjusted life years (QALY) lost over the first two months for all strategies as a function of the vaccination pace. **f** Relative reduction (RR) in occupied ICU beds at the peak over the first two months for all strategies as a function of the average daily number of first-dose vaccinations. We consider here the baseline scenario with intermediate vaccination coverage - i.e. same parameters as in Fig. 2d, e. In all panels, data are represented as means over 2000 independent stochastic realisations and error bars are derived from the standard error of the mean.

Supplementary Note 2: Combined reactive and mass vaccination for managing sustained COVID-19 spread

1. Additional results

In Supplementary Figure 8 we show the incidence curve corresponding to the scenarios analysed in Fig. 3a of the main paper. Mass and combined vaccination with three different vaccination paces are compared.

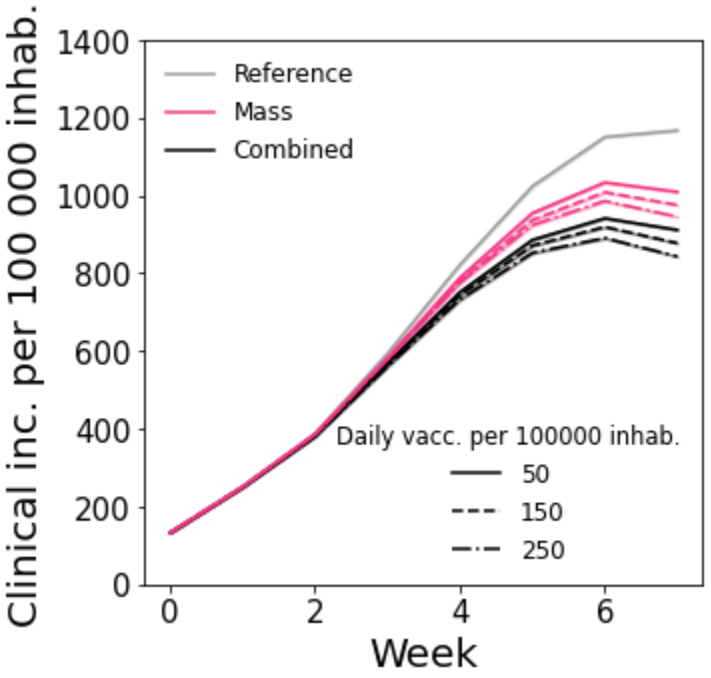

Supplementary Figure 8: Incidence of clinical cases for mass and combined vaccination strategies in the case of sustained spread. Scenarios are the same as the ones plotted in Fig. 3a of the main paper. Continuous lines are means over 2000 independent stochastic realisations and error bands are derived from the standard error of the mean (+/- 2SEM) - this is very low for this set of parameters.

Supplementary Note 3: Combined reactive and mass vaccination for managing a COVID-19 flare-up

1. Additional results

In Supplementary Figure 9 we show the incidence curve corresponding to the scenarios analysed in Fig. 4 of the main paper. Mass and combined vaccination with the different vaccination scenarios considered are compared.

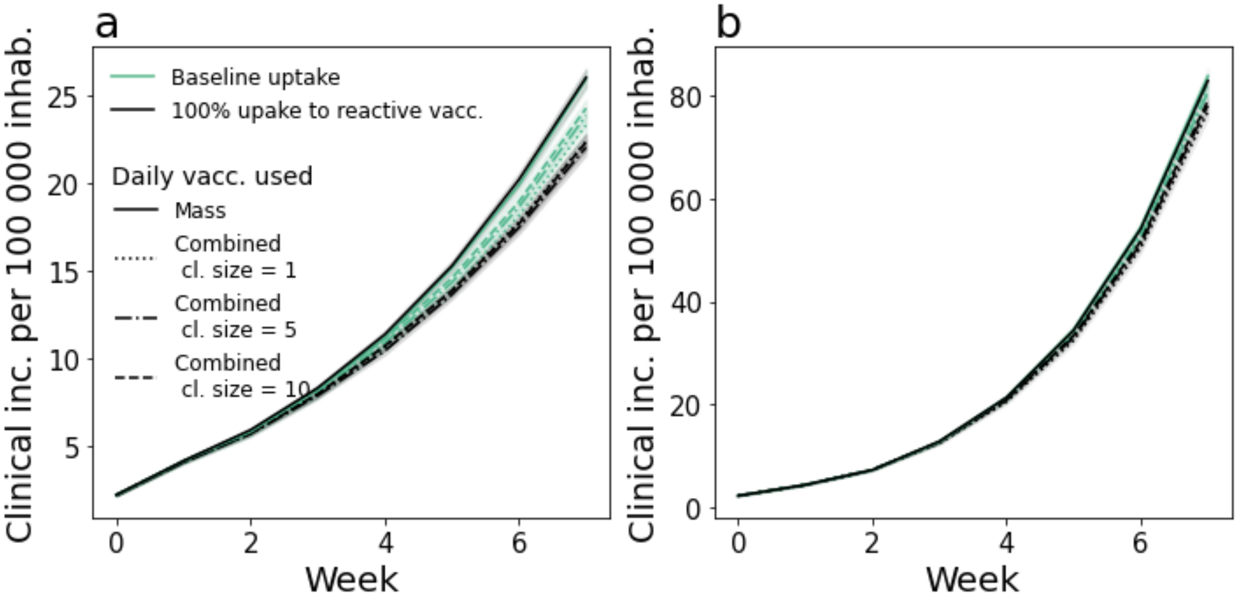

Supplementary Figure 9: Incidence of clinical cases for mass and combined vaccination strategies in the case of flare-up. Scenarios are the same as the ones plotted in Fig. 4 of the main paper. **a** Scenario with enhanced TTI. **b** Scenario with baseline TTI. In both panels, continuous lines are means over 8000 independent stochastic realisations and error bands are derived from the standard error of the mean.

2. Sensitivity analysis

In Supplementary Figure 10 we analyse the impact of combined and mass vaccination in a flare up scenario similarly to Fig. 4 of the main paper, by varying the hypotheses on virus transmissibility and vaccine escape. Specifically we consider values of the reproductive ratio from 1.2 to 1.8, and both worst and baseline vaccine effectiveness level - the worst vaccine effectiveness level is the same as in Fig. 2i of the main paper. Analogously to Fig. 4 we compare the attack rate for combined and mass vaccination, assuming both baseline and enhanced TTI and both baseline and 100% vaccine uptake in the context of reactive vaccination. We consider only the case in which reactive vaccination starts after the detection of the first case. For each set of parameters, scenarios with enhanced TTI and 100% uptake are associated with smaller attack rates and larger difference between mass and combined than the corresponding scenarios with baseline TTI and baseline uptake.

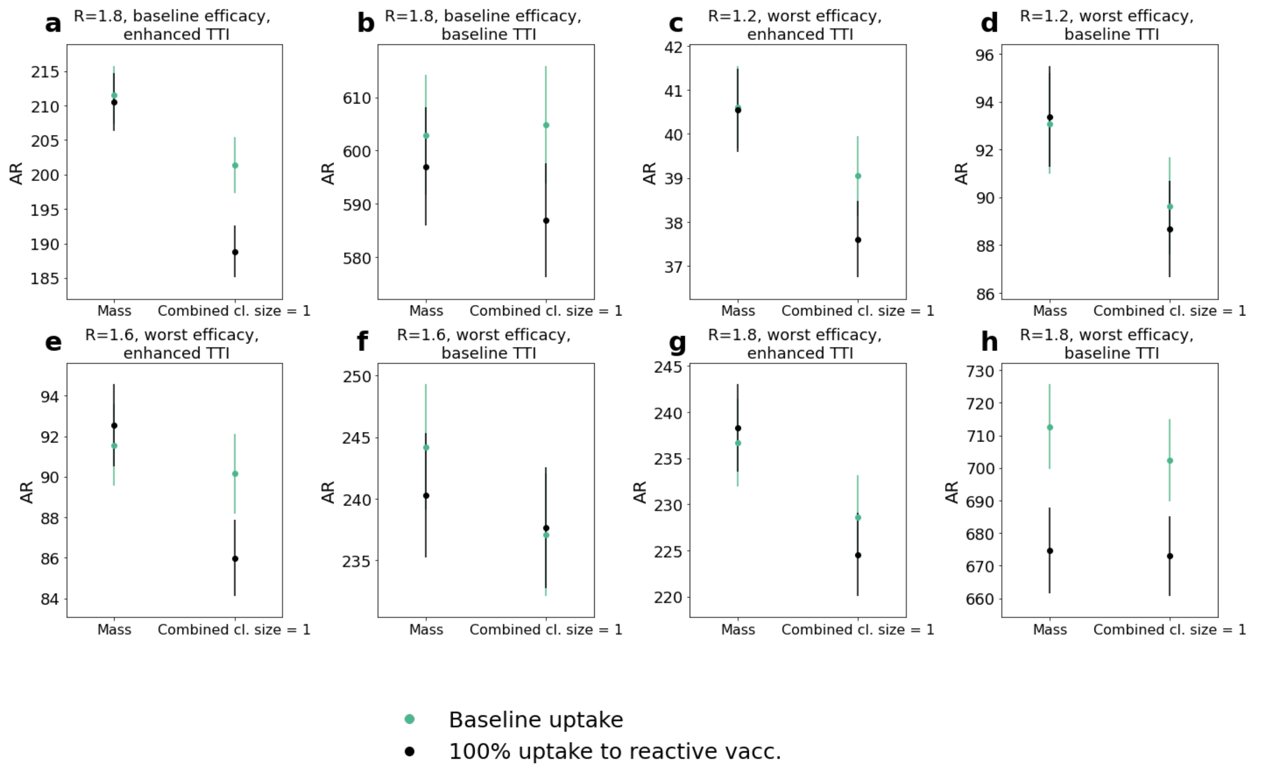

Supplementary Figure 10: Combined reactive and mass vaccination for managing a COVID-19 flare-up - sensitivity analysis. Average attack rate per 100000 inhabitants after two months for the flare-up case under different hypotheses: **a, b,** $R = 1.8$ with baseline vaccine effectiveness; **c-h** worst vaccine effectiveness with $R = 1.2$ (**c**,**d**), $R = 1.6$ (**e**,**f**) and $R = 1.8$ (**g**,**h**). The worst vaccine effectiveness scenario is defined as in Fig. 2 i of the main paper, i.e. $VE_{S,1} = 30\%$, $VE_{SP,1} = 35\%$, $VE_{S,2} = 53\%$ and $VE_{SP,2} = 60\%$. We compare enhanced and baseline TTI - a, c, e, g and b, d, f, h, respectively -, as well as baseline and 100% vaccine uptake. In all panels, parameters are the same as in Fig. 4 except for otherwise indicated. In all panels, data are represented as means over 8000 independent stochastic realisations and error bars are derived from the standard error of the mean.

We then investigate the impact of combined and mass vaccination on the extinction of the flare-up. In Supplementary Figure 11a we plot the probability of extinction for the scenario considered in Fig. 4a of the main paper (enhanced TTI and 100% vaccine uptake). We find that the probability of extinction is ~5%, and the difference between mass and combined is ~0.5%. The probability of extinction progressively increases under more optimistic hypotheses: increase in case detection from 70% and 30% (enhanced TTI) to 100% and 50% (strong TTI) for clinical and subclinical cases, respectively; increase in vaccine effectiveness to the best case scenario considered in Fig. 2i; rapid mounting of the vaccine effect, with partial immunity against infection already present one week after inoculation. In the best-case scenario plotted in panel h, the

probability of extinction reaches ~0.15 and ~0.18 for mass and combined vaccination, respectively.

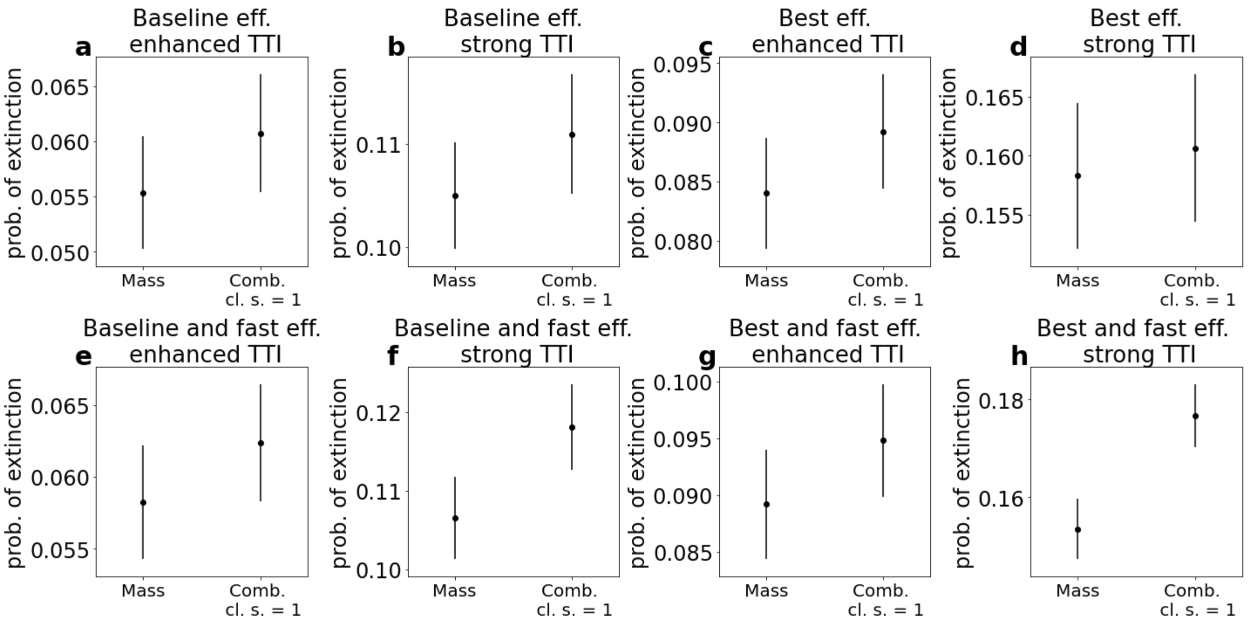

Supplementary Figure 11: Combined reactive and mass vaccination for managing a COVID-19 flare-up - probability of extinction. Three sets of parameters are investigated. 1) Baseline vaccine effectiveness (**a,b,e,f**) vs best effectiveness, i.e. $VE_{S,1} = 65\%$, $VE_{SP,1} = 75\%$,$VE_{S,2} = 80\%$ and $VE_{SP,2} = 95\%$, (**c,d,g,h);** 2) enhanced TTI (**a,c,e,g**) vs. strong TTI with $p_{d,c}$= 1 and $p_{d,sc}$= 0.5 (**b,d,f,h**). 3) 2 weeks (baseline) for vaccines to reach partial effectiveness (**a,b,c,d**) vs 1 week (**e,f,g,h**). In all panels, parameters are the same as in Fig. 4. In all panels, values represent the fraction of 15000 stochastic runs where the epidemic ends before two months. Error bars represent the standard error assuming the number of extinctions follows a binomial distribution.

Supplementary References

1. Moreno López, J. A. *et al.* Anatomy of digital contact tracing: Role of age, transmission setting, adoption and case detection. *Science Advances* eabd8750 (2021) doi:10.1126/sciadv.abd8750.
2. Kang, M. *et al. Transmission dynamics and epidemiological characteristics of Delta variant infections in China*. 2021.08.12.21261991 https://www.medrxiv.org/content/10.1101/2021.08.12.21261991v1 (2021) doi:10.1101/2021.08.12.21261991.

3. Lauer, S. A. *et al.* The Incubation Period of Coronavirus Disease 2019 (COVID-19) From Publicly Reported Confirmed Cases: Estimation and Application. *Ann Intern Med* **172**, 577–582 (2020).
4. Xin, H. *et al.* The Incubation Period Distribution of Coronavirus Disease 2019: A Systematic Review and Meta-analysis. *Clinical Infectious Diseases* (2021) doi:10.1093/cid/ciab501.
5. He, X. *et al.* Temporal dynamics in viral shedding and transmissibility of COVID-19. *Nature Medicine* 1–4 (2020) doi:10.1038/s41591-020-0869-5.
6. Di Domenico, L., Pullano, G., Sabbatini, C. E., Boëlle, P.-Y. & Colizza, V. Impact of lockdown on COVID-19 epidemic in Île-de-France and possible exit strategies. *BMC Medicine* **18**, 240 (2020).
7. Higdon, M. M. *et al. A systematic review of COVID-19 vaccine efficacy and effectiveness against SARS-CoV-2 infection and disease*. 2021.09.17.21263549 https://www.medrxiv.org/content/10.1101/2021.09.17.21263549v1 (2021) doi:10.1101/2021.09.17.21263549.
8. Polack, F. P. *et al.* Safety and Efficacy of the BNT162b2 mRNA Covid-19 Vaccine. *New England Journal of Medicine* **383**, 2603–2615 (2020).
9. Kissler, S. M. *et al.* Viral Dynamics of SARS-CoV-2 Variants in Vaccinated and Unvaccinated Persons. *New England Journal of Medicine* **385**, 2489–2491 (2021).
10. SPF. COVID-19 : point épidémiologique du 6 mai 2021. https://www.santepubliquefrance.fr/maladies-et-traumatismes/maladies-et-infections-respiratoires/infection-a-coronavirus/documents/bulletin-national/covid-19-point-epidemiologique-du-6-mai-2021.
11. Smith, L. E. *et al.* Adherence to the test, trace and isolate system: results from a time series of 21 nationally representative surveys in the UK (the COVID-19 Rapid Survey of Adherence to Interventions and Responses [CORSAIR] study). *medRxiv* (2020) doi:10.1101/2020.09.15.20191957.

12. Info Coronavirus COVID-19 - Tester - Alerter - Protéger. *Gouvernement.fr* https://www.gouvernement.fr/info-coronavirus/tests-et-depistage.

13. Nasreen, S. *et al. Effectiveness of mRNA and ChAdOx1 COVID-19 vaccines against symptomatic SARS-CoV-2 infection and severe outcomes with variants of concern in Ontario*. 2021.06.28.21259420 https://www.medrxiv.org/content/10.1101/2021.06.28.21259420v3 (2021) doi:10.1101/2021.06.28.21259420.

14. Tang, P. *et al.* BNT162b2 and mRNA-1273 COVID-19 vaccine effectiveness against the SARS-CoV-2 Delta variant in Qatar. *Nat Med* 1–8 (2021) doi:10.1038/s41591-021-01583-4.

15. Kiem, C. T. *et al.* Short and medium-term challenges for COVID-19 vaccination: from prioritisation to the relaxation of measures. (2021).

16. Lapidus, N. *et al.* Do not neglect SARS-CoV-2 hospitalization and fatality risks in the middle-aged adult population. *Infectious Diseases Now* **51**, 380–382 (2021).

17. Lefrancq, N. *et al.* Evolution of outcomes for patients hospitalised during the first 9 months of the SARS-CoV-2 pandemic in France: A retrospective national surveillance data analysis. *The Lancet Regional Health – Europe* **5**, (2021).

18. Davies, N. G. *et al.* Age-dependent effects in the transmission and control of COVID-19 epidemics. *Nature Medicine* 1–7 (2020) doi:10.1038/s41591-020-0962-9.

19. Sheikh, A., McMenamin, J., Taylor, B. & Robertson, C. SARS-CoV-2 Delta VOC in Scotland: demographics, risk of hospital admission, and vaccine effectiveness. *Lancet* **397**, 2461–2462 (2021).

20. Bager, P. and Wohlfahrt, J. and Rasmussen, M. and Albertsen, M and Grove Krause, T. Hospitalisation associated with SARS-CoV-2 delta variant in Denmark. *Lancet Infect Dis* (2021).

21. Salje, H. *et al.* Estimating the burden of SARS-CoV-2 in France. *Science* (2020) doi:10.1126/science.abc3517.

22. Tables de mortalité par sexe, âge et niveau de vie − Tables de mortalité par niveau de vie | Insee. https://www.insee.fr/fr/statistiques/3311422?sommaire=3311425.

23. Chevalier, J. & de Pouvourville, G. Valuing EQ-5D using time trade-off in France. *Eur J Health Econ* **14**, 57–66 (2013).
