# Supplementary material for: Agent-based modelling of reactive vaccination of workplaces and schools against COVID-19
Source: Nat Commun. 2022 Mar 17;13:1414. doi: 10.1038/s41467-022-29015-y (PMC8931017; doi:10.1038/s41467-022-29015-y)
Supplement: Supplementary file 3 — Reporting Summary [file 41467_2022_29015_MOESM3_ESM.pdf]

nature portfolio

Corresponding author(s): Chiara Poletto

Last updated by author(s): Feb 4, 2022

# Reporting Summary

Nature Portfolio wishes to improve the reproducibility of the work that we publish. This form provides structure for consistency and transparency in reporting. For further information on Nature Portfolio policies, see our Editorial Policies and the Editorial Policy Checklist.

## Statistics

For all statistical analyses, confirm that the following items are present in the figure legend, table legend, main text, or Methods section.

| n/a | Confirmed | |
|---|---|---|
| ☐ | ☒ | The exact sample size ($n$) for each experimental group/condition, given as a discrete number and unit of measurement |
| ☒ | ☐ | A statement on whether measurements were taken from distinct samples or whether the same sample was measured repeatedly |
| ☒ | ☐ | The statistical test(s) used AND whether they are one- or two-sided<br>*Only common tests should be described solely by name; describe more complex techniques in the Methods section.* |
| ☒ | ☐ | A description of all covariates tested |
| ☒ | ☐ | A description of any assumptions or corrections, such as tests of normality and adjustment for multiple comparisons |
| ☐ | ☒ | A full description of the statistical parameters including central tendency (e.g. means) or other basic estimates (e.g. regression coefficient) AND variation (e.g. standard deviation) or associated estimates of uncertainty (e.g. confidence intervals) |
| ☒ | ☐ | For null hypothesis testing, the test statistic (e.g. $F$, $t$, $r$) with confidence intervals, effect sizes, degrees of freedom and $P$ value noted<br>*Give P values as exact values whenever suitable.* |
| ☒ | ☐ | For Bayesian analysis, information on the choice of priors and Markov chain Monte Carlo settings |
| ☒ | ☐ | For hierarchical and complex designs, identification of the appropriate level for tests and full reporting of outcomes |
| ☒ | ☐ | Estimates of effect sizes (e.g. Cohen's $d$, Pearson's $r$), indicating how they were calculated |

*Our web collection on statistics for biologists contains articles on many of the points above.*

## Software and code

Policy information about availability of computer code

| | |
|---|---|
| Data collection | The synthetic population used as input for the computational model has been generated from open source data (available from insee.fr) as described in a previously published work (the corresponding citation is reported in the paper). |
| Data analysis | The code used for the simulations is written in C++11 compiled with g++ 5.2.0. It is available on zenodo at the link https://zenodo.org/record/5910314#.Yf1b-vXMI-T |

For manuscripts utilizing custom algorithms or software that are central to the research but not yet described in published literature, software must be made available to editors and reviewers. We strongly encourage code deposition in a community repository (e.g. GitHub). See the Nature Portfolio guidelines for submitting code & software for further information.

## Data

Policy information about availability of data

All manuscripts must include a data availability statement. This statement should provide the following information, where applicable:

- Accession codes, unique identifiers, or web links for publicly available datasets
- A description of any restrictions on data availability
- For clinical datasets or third party data, please ensure that the statement adheres to our policy

The synthetic population used in the analysis are available on zenodo at the link https://zenodo.org/record/5910314#.Yf1b-vXMI-T

# Field-specific reporting

Please select the one below that is the best fit for your research. If you are not sure, read the appropriate sections before making your selection.

☒ Life sciences ☐ Behavioural & social sciences ☐ Ecological, evolutionary & environmental sciences

For a reference copy of the document with all sections, see nature.com/documents/nr-reporting-summary-flat.pdf

# Life sciences study design

All studies must disclose on these points even when the disclosure is negative.

| | |
|---|---|
| Sample size | Analyses in Figure 2 and 3 of the main paper were obtained running 2000 stochastic simulations. This number was sufficiently large to lead to stable statistical estimates. Figure 4 was obtained by running 8000 stochastic simulations. In the parameter regime of Figure 4, the epidemic is close to extinction, thus stochastic fluctuations play an important role. For this reason an increased number of simulations was necessary. |
| Data exclusions | We only considered data for a mid-size french city and excluded census data not concerning the city.<br>No data was excluded from the simulation results to compute the summary measurements in Tables and Figures. |
| Replication | Simulation of each scenario was executed independently and with a different seed of the random number generator.<br>In Fig 2 & 3, each data point was based on 2000 replications. In Fig 4, each data point was based on 8000 replications.<br>All simulations yielded data that could be used in the analysis. |
| Randomization | We used the minimum standard congruential random generator (rminstd_rand0) as implemented in libstdc++ 5.2.0 to simulate random numbers in stochastic simulations.<br>No randomization was necessary in the investigation as we undertook a systematic numerical exploration of hypothetical scenarios. |
| Blinding | No blinding was necessary as the results of the simulations could not be affected by the investigator's knowledge of the scenarios under study. |

# Reporting for specific materials, systems and methods

We require information from authors about some types of materials, experimental systems and methods used in many studies. Here, indicate whether each material, system or method listed is relevant to your study. If you are not sure if a list item applies to your research, read the appropriate section before selecting a response.

## Materials & experimental systems

| n/a | Involved in the study | |
|---|---|---|
| ☒ | ☐ | Antibodies |
| ☒ | ☐ | Eukaryotic cell lines |
| ☒ | ☐ | Palaeontology and archaeology |
| ☒ | ☐ | Animals and other organisms |
| ☒ | ☐ | Human research participants |
| ☒ | ☐ | Clinical data |
| ☒ | ☐ | Dual use research of concern |

## Methods

| n/a | Involved in the study | |
|---|---|---|
| ☒ | ☐ | ChIP-seq |
| ☒ | ☐ | Flow cytometry |
| ☒ | ☐ | MRI-based neuroimaging |
